# National Latino AIDS Awareness Day — October 15, 2017

**DOI:** 10.15585/mmwr.mm6640a1

**Published:** 2017-10-13

National Latino AIDS Awareness Day is observed each year on October 15 to focus attention on the continuing disproportionate impact of human immunodeficiency virus (HIV) infection and acquired immunodeficiency syndrome (AIDS) on the Hispanic or Latino population in the United States. As of July 2015, the population of Hispanics or Latinos was estimated at 56.6 million, or approximately 18% of the U.S. population (*1*). However, in 2015, Hispanics or Latinos accounted for 24% of all new HIV diagnoses (*2*).

At the end of 2014, an estimated 235,600 Hispanics or Latinos were living with HIV infection in the United States. In 38 jurisdictions with complete reporting of CD4 and viral load data, 75.4% were linked to care within 1 month of diagnosis, 70.2% received HIV medical care, and 58.2% were virally suppressed (*3*).

National Latino AIDS Awareness Day is an opportunity to encourage increased HIV prevention activities, such as HIV testing, for Hispanics or Latinos. CDC supports testing, linkage to, and engagement in care and treatment, and a range of other efforts to reduce the risk for acquiring or transmitting HIV infection among Hispanics or Latinos. Additional information is available at https://www.cdc.gov/Features/LatinoAIDSAwareness.
